# Estimated Incidence and Factors Associated With Risk of Elder Mistreatment in New York State

**DOI:** 10.1001/jamanetworkopen.2021.17758

**Published:** 2021-08-02

**Authors:** David Burnes, David W. Hancock, John Eckenrode, Mark S. Lachs, Karl Pillemer

**Affiliations:** Factor-Inwentash Faculty of Social Work, University of Toronto, Toronto, Ontario, Canada (Burnes); Division of Geriatrics and Palliative Medicine, Weill Cornell Medicine, New York City, New York (Hancock, Lachs); Department of Human Development, Cornell University, Ithaca, New York (Eckenrode, Pillemer).

## Abstract

**IMPORTANCE:**

Elder mistreatment is associated with major health and psychosocial consequences and is recognized by clinicians, policy makers, and researchers as a pervasive problem affecting a rapidly aging global population.

**OBJECTIVE:**

To estimate the incidence of elder mistreatment and identify factors associated with the risk of new cases.

**DESIGN, SETTING, AND PARTICIPANTS:**

This research is a 10-year, longitudinal, population-based, cohort study of the incidence of elder mistreatment in New York State households conducted between 2009 (wave 1) and 2019 (wave 2). At wave 1, random digit-dial (landline and cellular telephones) stratified sampling was done to recruit English-speaking and/or Spanish-speaking, cognitively intact, community-dwelling older adults (aged ≥60 years) across New York State. The current study conducted computer-assisted telephone interviews with older adults who participated in wave 1 and gave permission to be contacted again for wave 2 interviews (response rate, 60.7%). Data analysis was performed from October 2020 to January 2021.

**EXPOSURES:**

Physical factors (health status, functional capacity, and age), living arrangement (coresidence), and sociocultural characteristics (sex, race/ethnicity, geocultural context, and household income).

**MAIN OUTCOMES AND MEASURES:**

Ten-year incidence for overall elder mistreatment and subtypes (financial abuse, emotional or psychological abuse, physical abuse, and neglect) were measured using adapted versions of the Conflict Tactics Scale, the Duke Older Americans Resources and Services scale, and the New York State Elder Mistreatment Prevalence Study financial abuse tool.

**RESULTS:**

The analytical sample included 628 older adults (mean [SD] age at wave 1, 69.20 [6.95] years; age at wave 2, 79.40 [6.93] years; 504 non-Hispanic White individuals [80.9%]; 406 women [64.6%]). Ten-year incidence rates were 11.4% (95% CI, 8.8%−14.3%) for overall elder mistreatment, 8.5% (95% CI, 6.3%–10.9%) for financial abuse, 4.1% (95% CI, 2.6%–5.7%) for emotional abuse, 2.3% (95% CI, 1.2%–3.6%) for physical abuse, and 1.0% (95% CI, 0.3%–1.8%) for neglect. Poor self-rated health at wave 1 was associated with increased risk at wave 2 of new overall mistreatment (odds ratio [OR], 2.86; 95% CI, 1.35–5.84), emotional abuse (OR, 3.67; 95% CI, 1.15–11.15), physical abuse (OR, 4.21; 95% CI, 1.14–13.70), and financial abuse (OR, 2.80; 95% CI, 1.16–6.38). Compared with non-Hispanic White participants, Black participants were at heightened risk of overall mistreatment (OR, 2.61; 95% CI, 1.16–5.70) and financial abuse (OR, 2.80; 95% CI, 1.09–6.91). A change from coresidence to living alone was associated with increased risk of financial abuse (OR, 2.74; 95% CI, 1.01–7.21).

**CONCLUSIONS AND RELEVANCE:**

These findings suggest that health care visits may be important opportunities to detect older adults who are at risk of mistreatment. Race is highlighted as an important social determinant for elder mistreatment requiring urgent attention.

## Introduction

Elder mistreatment (EM) refers to an intentional act or lack of action by a person in a relationship involving an expectation of trust that causes harm or risk of harm to an older adult. It comprises 5 subtypes, including financial abuse or exploitation, emotional or psychological abuse, physical abuse, sexual abuse, and neglect by others.^1^ EM is recognized by clinicians, policy makers, and researchers as a pervasive problem with serious consequences affecting a rapidly aging global population.^1–3^ Specifically, the Centers for Disease Control and Prevention recently defined EM as a serious public health issue requiring formal surveillance,^1^ and the 2015 decennial White House Conference on Aging designated EM as 1 of 4 top-priority issues affecting older adults.^2^

Recent reviews^4,5^ of population-based EM studies have found 1-year period prevalence rates of 15.7% globally and 9.5% in the US among cognitively intact, community-dwelling older adults. EM is associated with serious health and psychosocial consequences, such as premature mortality, poor physical and mental health, diminished quality of life, and increased rates of emergency services use, hospitalization, and nursing home placement.^6–8^ Unfortunately, an understanding of effective community-based EM prevention strategies represents the largest knowledge gap in the literature^5^; systematic reviews routinely find that existing prevention programs are informed by weak research evidence.^9,10^

The development of effective prevention strategies is predicated on understanding the factors associated with risk of EM. Knowledge of these factors is required to develop targeted primary prevention efforts, such as EM screening tools or public education and awareness efforts, designed to forestall the occurrence of EM. In the absence of reliable screening instruments, for example, health care practitioners underdetect older adults who are at high risk of EM.^11^ Accurate knowledge of these factors also contributes to the development of evidence-based, secondary prevention interventions.^12^

Basic research on the factors associated with the risk of EM has generally been limited to the use of convenience sample research designs, often drawing research participants from social service or clinical health settings,^13–16^ which carry selection bias and threats to external validity. Initial explorations of factors associated with risk of EM emerged from geographically confined, prospective studies that linked population-based older adult samples to Adult Protective Services program records as a means to identify individuals experiencing EM.^8,17,18^ However, EM cases referred to Adult Protective Services constitute a very small and potentially biased subsample of individuals experiencing EM in the general population.^19^ Finally, regional, state, or national population-based cross-sectional EM studies have generated representative information about the prevalence and correlates of EM with greater external validity.^20–24^ However, the cross-sectional design of these population-based studies violates the temporal association between proposed risk factors and the occurrence of EM. Also, without establishing baseline EM status among cases, it is not possible to identify factors associated with the risk of new cases emerging over time.

For these reasons, there is a pressing need for incidence studies of EM. Incidence research is invaluable in understanding factors associated with the incidence of EM because varying rates among subgroups with different exposures can be calculated. To date, population-based EM studies have focused on problem prevalence, typically over a 1-year period. However, prevalence alone does not provide information about the rate of new cases entering the population over time and does not allow identification of factors associated with increased risk of new EM cases among older adults. Prior research on EM incidence has used retrospective, case record, convenience sampling, and/or sentinel-based (as opposed to direct interviewing) data collection designs.^25–27^ A recent prospective EM incidence study^13^ found a 2-year incidence rate of 8.8%, which used a sampling approach through community centers in a single urban area (Chicago, Illinois) and was limited to Chinese older adults.

Stemming from the New York State Elder Mistreatment Prevalence Study (NYSEMPS),^28^ the current study advances prior EM incidence research using a longitudinal, population-based design to estimate an incidence rate and identify factors associated with the risk of EM occurring over time. Guided by the ecologically based EM risk framework proposed by the National Research Council,^29^ this study hypothesized that older adults with higher levels of physical vulnerability, coresidence, and sociocultural disadvantage would be at heightened risk of experiencing EM over the course of 10 years.

## Methods

### Sample

This cohort study received ethics approval from the University of Toronto, Weill Cornell Medicine, and Cornell University. Participants consented orally during the telephone-based interview. This study follows the Strengthening the Reporting of Observational Studies in Epidemiology (STROBE) reporting guideline for cohort studies.

The NYSEMPS used a random digit-dial (landline and cellular telephones) stratified sampling strategy derived from US Census tracts of New York State in 2009 to conduct telephone interviews with a representative (race/ethnicity and sex) sample of 4156 older adults (wave 1).^28^ Inclusion criteria were age 60 years or older, English-speaking and/or Spanish-speaking, community dwelling (ie, not living in an institutional setting), and cognitively intact, as determined by a modified version of the Abbreviated Mental Test.^30^ To avoid exclusion of older adults with potentially high risk of EM, proxy interviews were conducted in 156 cases when the older adult had physical, communication, or language barriers preventing direct interviewing.

Among the wave 1 sample, 2964 respondents (71.3%) provided permission to be recontacted for a follow-up study. Recontact permission status did not vary significantly according to sex or race/ethnicity, although respondents providing recontact permission were younger than those who did not provide permission (mean [SD] age, 73.7 [8.7] years vs 75.1 [8.6] years). Approximately 10 years later, in 2019 (wave 2), computer-assisted telephone interviews were conducted by the University of New Hampshire Survey Center with 628 older adults who provided recontact permission. Of the 2964 older adults providing recontact permission, 1548 (52.2%) were ineligible because of death (946 individuals [31.9%]) or because they were living in a nursing home or had a physical inability to participate with no proxy available (602 individuals [20.3%]). Using American Association for Public Opinion Research criteria, wave 2 data collection achieved a 60.7% response rate and 77.9% cooperation rate (eTable 1 in the Supplement).

Efforts to recruit wave 2 participants were comprehensive. Informational letters were mailed to all prospective participants in advance of receiving a telephone call to both cue recall of wave 1 participation and prime them for potential wave 2 interviews. Search engine and directory-matching services (eg, White Pages Premium and Lexis-Nexis) were used to track participants with out-of-date contact information. A strong call-outreach effort was implemented to contact participants, including up to 20 call-back attempts across varying days and times of the week. Prospective participants also received follow-up mailed letters and were offered a $50 electronic Amazon gift card.

### Dependent Variable

Ten-year EM incidence was defined by cases reporting EM since age 60 years at wave 2 (2019) who reported no EM history since age 60 years at wave 1 (2009). This allowed us to isolate those older adults who experienced EM between 2009 and 2019. Older adults who reported EM history at wave 1 were removed from our analytical sample to ensure that the sample only included new cases over the 10-year study period.

Well-established measures were adapted to assess emotional, physical, and sexual abuse and neglect. A modified version of the Conflict Tactics Scale (CTS)^5,20,23^ was used to assess elder emotional, physical, and sexual abuse. Emotional abuse was assessed using 3 CTS items related to spiteful behavior, insulting or swearing, and threatening to hit or throw something. Physical abuse was assessed using 11 CTS items related to throwing, hitting, slapping, pushing, grabbing, kicking, biting, beating, and using or threatening to use a knife or gun. Sexual abuse was assessed with 2 CTS items related to unconsented touching or intercourse. Elder neglect was evaluated by failure to meet an older adult’s needs by a responsible caregiver, using the Duke Older Americans Resources and Services (OARS) instrumental activities of daily living (IADL) and ADL scales covering functional capacity related to shopping, meal preparation, housework, taking medication, cutting or eating food, dressing or undressing, walking, getting in or out of bed, bathing or showering, and using the bathroom.^5,31^

In the absence of an adequate financial abuse measure at wave 1 validated for use with general population-based older adult samples, the NYSEMPS developed a 5-item financial abuse tool.^28^ The tool draws on existing measures,^32,33^ and items were developed using a 2-stage consensus process involving researchers in financial abuse and clinicians.^28^ The tool covers scenarios related to stolen or misappropriated money or property; coercion or false pretense resulting in surrendering rights, property, or signing or changing a legal document; impersonation to obtain property or services; and inadequate contributions toward household or basic expenses. The items were piloted in a small group of 10 individuals known to have experienced financial abuse; all pilot participants tested positive. Consistent with recommendations to maximize sensitivity in epidemiological interpersonal violence research,^29^ the CTS, Duke OARS, and financial abuse tools assessed EM subtypes with multiple contextually oriented, behaviorally defined items describing specific mistreatment events.

EM incidence was measured dichotomously as the absence vs presence of emotional, physical, sexual, or financial abuse or neglect. Across EM subtype measures, respondents were asked whether they had experienced specific mistreatment behaviors (items) since age 60 years. Affirmative responses initiated a follow-up appraisal question about the level of seriousness (not serious, somewhat serious, or very serious) attached to the mistreatment.^28^ Physical, sexual, and financial abuse cases were deemed positive if an item was affirmed regardless of perceived seriousness. In accordance with recent recommendations to enhance EM measurement specificity,^5^ emotional abuse cases were deemed positive if the spiteful behavior or insulting or swearing CTS items were rated as very serious or if the threat to hit or throw CTS item was affirmed with any level of seriousness. Similarly, neglect cases were assessed as positive if any of the Duke OARS ADL or IADL unmet needs were appraised by the respondent as somewhat or very serious. Consistent with accepted EM definitions,^1,29^ this study restricted elder abuse or neglect to scenarios occurring in relationships involving an expectation of trust.

### Independent Variables

Physical vulnerability was represented by functional impairment, self-reported health status, and age. Functional impairment was measured continuously as the number of Duke OARS ADL or IADL tasks requiring assistance (range, 0–10 tasks). A functional impairment change score variable was constructed (no change, increased impairment, or decreased impairment) to reflect any change in functional status that occurred between wave 1 and wave 2. Self-reported health status was measured dichotomously as poor (very poor, poor, or fair) or good (good, very good, or excellent); a self-reported health wave 1 to wave 2 change score was also constructed (no change, health decline, or health improvement). Age was measured continuously.

Coresidence status was measured dichotomously according to whether an older adult lived alone or with others; a wave 1 to wave 2 coresidence change score was constructed to reflect whether an older adult experienced no change in living arrangement, moved from living alone to living with others, or moved from living with others to living alone. Self-reported sociocultural characteristics based on predefined response options included sex (male or female), race/ethnicity (non-Hispanic White, African American or Black, Hispanic White, American Indian Aleut or Eskimo, Asian or Pacific Islander, or other [open field]), and household income. Race/ethnicity data were collected to understand whether EM incidence and vulnerability differ across older adult racial/ethnic subgroups. Mean annual household income was measured continuously to reflect 9 sequential income categories (<$10 000; $10 000 to <$20 000; $20 000 to <$30 000; $30 000 to <$40 000; $40 000 to <$50 000; $50 000 to <$75 000; $75 000 to <$100 000; $100 000 to <$150 000; and ≥$150 000); a household income wave 1 to wave 2 change score was also constructed (no income change, declined income, or increased income). Geographical context was defined by urban, suburban, or rural environment, as determined by the New York State Office of Mental Health.^28^

### Statistical Analysis

All analyses first examined the presence of any EM, followed by analyses examining subtypes of EM. Two-sided χ^2^ or *t* tests, as appropriate, were used to compare which participants were likely to participate in the follow-up survey. Models controlled for characteristics demonstrating significant differences between those who did and did not participate in the follow-up survey. Binary logistic regression analyses examined whether hypothesized independent variables at wave 1 were associated with the incidence of EM at wave 2. For any separation in the logistic regression models, the Firth bias reduction method, which produces estimates based on a penalized maximum likelihood estimation, was used to estimate the model, using the R package logistf.^34,35^ Finally, as an exploratory analysis, binary logistic regressions examined the association between incidence of EM at wave 2 and categorical change scores of the independent variables from wave 1 to wave 2. Missing data were deleted listwise on analytical variables (eTable 2 in the Supplement). There were no significant differences between groups with and without missing data for any of the analytical variables. A significance level of *P* ≤ .05 was used in analyses. Analyses were conducted in SPSS statistical software version 23 (IBM) and R Studio statistical software version 1.3.959 (R Project for Statistical Computing). Data analysis was performed from October 2020 to January 2021.

## Results

### Descriptive Statistics

Among the 628 sample participants (mean [SD] age at wave 1, 69.20 [6.95] years; age at wave 2, 79.40 [6.93] years), most were women (406 women [64.6%]) and non-Hispanic White (504 participants [80.9%]). Most participants reported good health (563 participants [89.6%]) and functional ADL or IADL independence (mean [SD] functional impairment score, 0.15 [0.69]), and they reported a median (interquartile range) household income category of less than $75 000 (<$30 000 to <$100 000) (Table 1). The following characteristics were associated with a significantly greater likelihood of participating in the wave 2 survey: non-Hispanic White race/ethnicity (504 participants [80.9%] [95% CI, 78.0% to 83.9%] participated in wave 2 vs 2501 participants [70.9%] [95% CI, 70.1% to 73.0%] who did not participate in wave 2), higher annual income (median [interquartile range], <$75 000 [<$30 000 to <$100 000] [95% CI, <$50 000 to <$75 000] among those who participated in wave 2 vs <$40 000 [<$20 000 to <$75 000] [95% CI, <$40 000 to <$40 000] among those who did not participate in wave 2), younger age (mean [SD] age, 69.22 [6.95] [95% CI, 68.66 to 69.78] years among those who participated in wave 2 vs 74.94 [8.68] [95% CI, 74.64 to 75.25] years among those who did not participate in wave 2), self-reported good health (563 participants [89.6%] [95% CI, 87.0% to 91.8%] who participated in wave 2 vs 2561 participants [72.7%] [95% CI, 71.2% to 74.1%] who did not participate in wave 2), and greater functional capacity (mean [SD] functional impairment score, 0.15 [0.69] [95% CI, 0.09 to 0.20] among those who participated in wave 2 vs 0.47 [1.30] [95% CI, 0.43 to 0.52] among those who did not participate in wave 2) (Table 2).

### Incidence Analyses

Incidence rates are presented in Table 3. The overall 10-year incidence of EM was 11.4% (95% CI, 8.8%–14.3%). The incidence rates of EM subtypes were 8.5% (95% CI, 6.3%–10.9%) for financial abuse, 4.1% (95% CI, 2.6%–5.7%) for emotional abuse, 2.3% (95% CI, 1.2%–3.6%) for physical abuse, 1.0% (95% CI, 0.3%–1.8%) for neglect, and 0.0% for sexual abuse.

Table 4 presents results of multivariable logistic regression analyses examining the associations of wave 1 independent variables with EM incidence at wave 2. Poor self-rated health at wave 1 was associated with increased risk of any new EM at wave 2 (odds ratio [OR], 2.86; 95% CI, 1.35–5.84; *P* = .004). For the different EM subtypes, poor self-rated health at wave 1 was associated with greater risk of emotional (OR, 3.67; 95% CI, 1.15–11.15; *P* = .03), physical (OR, 4.21; 95% CI, 1.14–13.70; *P* = .03), and financial (OR 2.80; 95% CI, 1.16–6.38; *P* = .02) abuse. Older age at wave 1 was associated with a greater risk of neglect at wave 2 (OR, 1.17; 95% CI, 1.04–1.38; *P* = .01). Finally, compared with non-Hispanic White participants, Black participants were significantly more likely to experience any EM (OR, 2.61; 95% CI, 1.16–5.70; *P* = .02), particularly financial abuse (OR, 2.80; 95% CI, 1.09–6.91; *P* = .03).

A final exploratory set of analyses examined whether changes in the variables of interest, rather than their wave 1 status alone, were associated with greater EM incidence (eTable 3, eTable 4, eTable 5, and eTable 6 in the Supplement). When controlling for the state of a variable at wave 1, the change in a variable between wave 1 and wave 2 was generally not associated with greater EM incidence. However, there was 1 notable exception related to change in living situation. Individuals who were living with others at wave 1 and moved to living alone at wave 2 were more likely to experience new EM of any type (OR, 2.27; 95% CI, 1.00–5.00; *P* = .01) and financial abuse (OR, 2.74; 95% CI, 1.01–7.21; *P* = .05) specifically.

## Discussion

This cohort study estimated the 10-year incidence of EM and identified factors associated with the risk of new cases entering the population over this period. Building on prior population-based research of factors associated with the risk of EM using cross-sectional designs, this longitudinal study advances our understanding of risk factors with enhanced rigor, including an ability to disentangle risk factors from potential consequences of EM.

An incidence rate of 11.4% indicates that approximately one-tenth of older adults living in New York State will experience EM over a 10-year period, which translates to more than 360 000 individuals. This incident rate is similar to the 1-year prevalence rate of 9.5% found across population-based studies in the US.^5^ Together, these findings suggest that approximately 1 in 10 older adults are either experiencing EM at any given time or will experience EM for the first time over a 10-year period. Given the scope of this issue, the development of prevention programs that either forestall initial onset of EM among older adults or support those already experiencing EM are urgently needed.

The development of such prevention programs requires knowledge about factors associated with the risk of EM. In the current study, poor health status in particular was significantly associated with overall EM and nearly all mistreatment subtypes. These findings bolster results from other research reporting an association between health and EM.^5,20–23^ The implication of these findings is that health care practitioners can, therefore, play a key role in screening, providing education and awareness, and/or making appropriate referrals for at-risk older adults. A validated screening tool would provide an opportunity to identify at-risk older adults and initiate interventions or supports before EM occurs.

Beyond health status, our study found that older adults who live alone are at increased risk of financial abuse. This finding challenges conventional thought in the field that shared living increases EM risk^5,29^ and indicates the possibility that living arrangement confers divergent risk depending on the EM subtype. Living alone may be associated with increased risk of financial abuse, specifically if a protective spouse or partner is not present to help manage household finances and others external to the household are required to access personal accounts. Results from the current study also revealed that Black older adults are at increased risk of financial abuse compared with White individuals, which is consistent with some prior research examining correlates of EM prevalence.^22,36^ This finding highlights an important form of racial disparity requiring urgent attention, particularly when situated in combination with the fact that Black older adults are disproportionately represented in the population experiencing poverty.^37^

### Limitations

This study has limitations that should be addressed. Reported incidence rates of EM likely underestimate the true population incidence because older adults tend to underreport personal problems.^38^ Potentially important factors associated with risk of EM or confounders were unavailable for analysis, including characteristics of individuals experiencing EM (eg, previous trauma, mental health status, or social support) and those of other trusted individuals (eg, psychiatric or mental health status, substance use, and economic dependence). Findings from this study cannot necessarily be generalized beyond New York State.

## Conclusions

To our knowledge, this cohort study is the first longitudinal, population-based, incidence study of EM. Our findings confirm what researchers, policy makers, and advocates have increasingly argued: EM is pervasive and only a small minority of cases are observed in service systems. The development and evaluation of EM prevention programs is critically needed to address risk of EM associated with health status, living arrangement, and race/ethnicity.

## Supplementary Material

Supplemental Tables **eTable 1.** American Association for Public Opinion Research (AAPOR) Sample Disposition and Calculation of Response/Cooperation Rates
**eTable 2.** Missing Data Analysis
**eTable 3.** Multivariable Logistic Regression for Change in Health Status Predicting Elder Mistreatment Incidence
**eTable 4.** Multivariable Logistic Regression for Change in Income Predicting Elder Mistreatment Incidence
**eTable 5.** Multivariable Logistic Regression for Change in Functional Capacity Predicting Elder Mistreatment Incidence
**eTable 6.** Multivariable Logistic Regression for Change in Living Arrangements

## Figures and Tables

**Table 1.** Descriptive Statistics at Wave 1 and Wave 2 of Analytical Sample

Characteristic	Participants, No. (%) (N = 628)^a^	
	Wave 1	Wave 2
Age, mean (SD), y	69.20 (6.95)	79.40 (6.93)
Sex		
Male	222 (35.4)	222 (35.4)
Female	406 (64.6)	406 (64.6)
Self-rated health		
Good	563 (89.6)	511 (81.5)
Poor	65 (10.4)	116 (18.5)
Functional impairment score, mean (SD)^b^	0.15 (0.69)	0.79 (1.74)
Race/ethnicity		
Non-Hispanic White	504 (80.9)	504 (80.9)
Non-Hispanic Black	78 (12.5)	78 (12.5)
Hispanic White	25 (4.0)	25 (4.0)
Other race/ethnicity^c^	16 (2.6)	16 (2.6)
Annual household income, median (interquartile range), $	<75 000 (<30 000 to <100 000)	<50 000 (<20 000 to <75 000)
Living situation		
Alone	205 (32.6)	264 (42.0)
With others	422 (67.2)	364 (58.0)
Geographical context^d^		
Urban	340 (54.1)	NA
Suburban	168 (26.8)	NA
Rural	118 (18.8)	NA

Abbreviation: NA, not assessed.

a Some percentages may not equal 100 because of missing data or the option to select multiple responses (for race).

b Functional impairment is a count measure of activities of daily living or instrumental activities of daily living with which the participant needs assistance (range, 0–10).

c Other refers to American Indian Aleut or Eskimo, Asian or Pacific Islander, or self-described other race/ethnicity.

d Geographical context was not reassessed at wave 2.

**Table 2.** Comparison of Those Who Participated at Wave 2 vs Those Who Did Not Participate at Wave 2 on Variables Measured at Wave 1

Characteristic	Participants, No. (%) [95% CI]^a^	
	Wave 2 participation	No wave 2 participation
Age, mean (SD) [95% CI], y^b^	69.22 (6.95) [68.66 to 69.78]	74.94 (8.68) [74.64 to 75.25]
Sex		
Male	222 (35.4) [31.7 to 39.2]	1254 (35.5) [34.0 to 37.1]
Female	406 (64.6) [60.8 to 68.3]	2274 (64.5) [62.9 to 66.0]
Self-rated health^b^		
Good	563 (89.6) [87.0 to 91.8]	2561 (72.7) [71.2 to 74.1]
Poor	65 (10.4) [8.2 to 13.0]	964 (27.3) [25.9 to 28.8]
Functional impairment score, mean (SD) [95% CI]^b,c^	0.15 (0.69) [0.10 to 0.20]	0.47 (1.30) [0.40 to 0.50]
Race/ethnicity^b^		
Non-Hispanic White	504 (80.9) [78.0 to 83.9]	2501 (70.9) [70.1 to 73.0]
Non-Hispanic Black	78 (12.5) [9.6 to 15.6]	679 (19.2) [17.9 to 20.9]
Hispanic White	25 (4.0) [1.1 to 7.0]	225 (6.4) [5.0 to 7.9]
Other race/ethnicity^d^	16 (2.6) [0.0 to 5.6]	91 (2.6) [1.1 to 4.1]
Annual household income, median (interquartile range) [95% CI], $^b^	<75 000 (<30 000 to <100 000) [<50,000 to <75,000]	<40 000 (<20 000 to <75 000) [<40 000 to <40 000]
Living situation		
Alone	205 (32.6) [29.1 to 36.5]	1368 (38.8) [37.4 to 40.7]
With others	422 (67.2) [63.5 to 70.9]	2136 (60.5) [59.3 to 62.6]
Geographical context		
Urban	340 (54.1) [50.3 to 58.5]	2055 (58.2) [56.7 to 60.1]
Suburban	168 (26.8) [22.8 to 31.0]	949 (26.9) [25.3 to 28.7]
Rural	118 (18.8) [14.9 to 23.0]	517 (14.7) [13.0 to 16.4]

a Some percentages may not equal 100 because of missing data, or the option to select multiple responses (race).

b There was a significant difference between groups.

c Functional impairment is a count measure of activities of daily living or instrumental activities of daily living with which the participant needs assistance (range, 0–10).

d Other refers to American Indian Aleut or Eskimo, Asian or Pacific Islander, or self-described other race/ethnicity.

**Table 3.** Incidence of Elder Mistreatment

Type of mistreatment	Bootstrapped incidence per 100 individuals (95% CI) (N = 10 000)
Any mistreatment	11.4 (8.8–14.3)
Financial	8.5 (6.3–10.9)
Emotional	4.1 (2.6–5.7)
Physical	2.3 (1.2–3.6)
Neglect	1.0 (0.3–1.8)
Sexual	0.0

**Table 4.** Multivariable Logistic Regression of Elder Mistreatment Incidence^a^

Independent variable	OR (95% CI)				
	Any mistreatment	Neglect	Emotional abuse	Physical abuse	Financial abuse
Age	1.02 (0.98–1.06)	1.17 (1.04–1.38)^b^	0.97 (0.89–1.04)	1.02 (0.94–1.10)	1.02 (0.97–1.07)
Female sex (reference, male)	0.63 (0.35–1.15)	1.19 (0.21–11.55)	0.47 (0.18–1.21)	0.37 (0.11–1.17)	0.73 (0.35–1.55)
Self-rated poor health (reference, good)	2.86 (1.35–5.84)^c^	1.73 (0.09–17.55)	3.67 (1.15–11.15)^d^	4.21 (1.14–13.70)^d^	2.80 (1.16–6.38)^e^
Annual household income	0.96 (0.82–1.13)	0.91 (0.53–1.49)	0.90 (0.69–1.17)	1.17 (0.87–1.61)	0.88 (0.73–1.06)
Race/ethnicity (reference, non-Hispanic White)					
Non-Hispanic Black	2.61 (1.16–5.70)^e^	4.55 (0.43–43.04)	1.35 (0.31–4.63)	1.15 (0.12–6.04)	2.80 (1.09–6.91)^d^
Hispanic White	0.74 (0.11–2.87)	2.97 (0.02–48.62)	0.42 (0.00–3.71)	0.60 (0.00–5.11)	1.33 (0.19–5.47)
Other race/ethnicity^f^	1.32 (0.20–5.15)	5.36 (0.04–74.33)	0.46 (0.00–4.88)	1.02 (0.01–8.80)	2.35 (0.34–9.62)
Geocultural context (reference, urban)					
Suburban	0.80 (0.39–1.56)	1.14 (0.10–9.80)	1.03 (0.32–2.98)	0.64 (0.15–2.12)	0.70 (0.26–1.69)
Rural	0.65 (0.26–1.48)	1.74 (0.14–18.35)	0.69 (0.16–2.33)	0.44 (0.04–2.14)	0.83 (0.28–2.18)
Lives with family (reference, lives alone)	0.74 (0.41–1.38)	0.91 (0.14–6.45)	1.32 (0.47–4.24)	0.46 (0.14–1.51)	0.64 (0.31–1.32)
Functional impairment	0.88 (0.53–1.26)	0.95 (0.48–1.56)	0.92 (0.02–1.60)	0.99 (0.02–1.71)	0.99 (0.62–1.40)
Observations, No.	523	515	509	521	516

Abbreviation: OR, odds ratio.

a Incidence was calculated using Firth bias reduced logistic regression. Model fit was assessed with Tjur *r*^2^, with values ranging from 0.03 to 0.19.

b *P* = .01.

c *P* = .004.

d *P* = .03.

e *P* = .02.

f Other refers to American Indian Aleut or Eskimo, Asian or Pacific Islander, or self-described other race/ethnicity.

## Key Points

### Question

What is the incidence of elder mistreatment (EM), and what factors are associated with new cases entering the population?

### Findings

In this cohort study including 628 older adults, the 10-year EM incidence rate was 11.4%. Poor health status was significantly associated with new cases of overall EM and financial, emotional and psychological, and physical abuse subtypes; Black race and living alone were significantly associated with overall EM and financial abuse specifically.

### Meaning

These findings suggest that health care visits may be important opportunities to detect at-risk older adults, and race is highlighted as a social determinant of EM requiring urgent attention.
