# Supplementary material for: Estimated Incidence and Factors Associated With Risk of Elder Mistreatment in New York State
Source: JAMA Netw Open. Author manuscript; Available in PMC 2022 Apr 18. (PMC9014652; doi:10.1001/jamanetworkopen.2021.17758)
Supplement: Supplemental Tables — eTable 1. American Association for Public Opinion Research (AAPOR) Sample Disposition and Calculation of Response/Cooperation Rates eTable 2. Missing Data Analysis eTable 3. Multivariable Logistic Regression for Change in Health Status Predicting Elder Mistreatment Incidence eTable 4. Multivariable Logistic Regression for Change in Income Predicting Elder Mistreatment Incidence eTable 5. Multivariable Logistic Regression for Change in Functional Capacity Predicting Elder Mistreatment Incidence eTable 6. Multivariable Logistic Regression for Change in Living Arrangements [file NIHMS1791147-supplement-Supplemental_Tables.pdf]

Supplemental Online Content

Burnes D, Hancock DW, Eckenrode J, Lachs MS, Pillemer K. Estimated incidence and factors associated with risk of elder mistreatment in New York State. *JAMA Netw Open*. 2021;4(8):e2117758. doi:10.1001/jamanetworkopen.2021.17758

eTable 1. American Association for Public Opinion Research (AAPOR) Sample Disposition and Calculation of Response/Cooperation Rates

eTable 2. Missing Data Analysis

eTable 3. Multivariable Logistic Regression for Change in Health Status Predicting Elder Mistreatment Incidence

eTable 4. Multivariable Logistic Regression for Change in Income Predicting Elder Mistreatment Incidence

eTable 5. Multivariable Logistic Regression for Change in Functional Capacity Predicting Elder Mistreatment Incidence

eTable 6. Multivariable Logistic Regression for Change in Living Arrangements

This supplemental material has been provided by the authors to give readers additional information about their work.

© 2021 Burnes D et al. *JAMA Network Open.*

eTable 1. American Association for Public Opinion Research (AAPOR) Sample Disposition and Calculation of Response/Cooperation Rates

Sample Dispositions	Pretest	Overall
	N (%)	N (%)
I=Complete Interviews	15 (14)	628 (21)
P=Partial Interviews	0 (0)	1 (0)
R=Refusal and break off	5 (5)	178 (6)
NC=Non Contact	0 (0)	6 (0)
O=Other	4 (4)	20 (1)
Calculated e*	0.289	0.35
UH=Unknown Household	22 (20)	452 (15)
UO=Unknown other	4 (4)	131 (4)
Not Eligible+	59 (54)	1548 (52)
Total Sample Used	109 (100)	2964 (100)

Response Rate: *(I+P)/((I+P) + (R+NC+O) + e(UH+UO));* Cooperation rate: *(I+P)/((I+P)+R));* * e is the estimated proportion of cases of unknown eligibility that are eligible. This estimate is based on the proportion of eligible units among all units in the sample for which a definitive determination of status was obtained (a conservative estimate); + Includes deceased, not physically able (and no available proxy), and those in a nursing home.

© 2021 Burnes D et al. *JAMA Network Open.*

eTable 2. Missing Data Analysis

Variable	% Missing Data
Age	0
Sex	0
Self-rated Health	0
Household Income	5.7
Race/Ethnicity	0.89
Geo-cultural Context	0.36
Co-Residence	0.18
Functional Impairment	0
Any Abuse at T2	0
Neglect at T2	1.6
Physical Abuse at T2	0.4
Emotional Abuse at T2	2.5
Financial Abuse at T2	1.2

There were no significant differences between groups with and without missing data (all $p > .05$).

© 2021 Burnes D et al. *JAMA Network Open.*

eTable 3. Multivariable Logistic Regression for Change in Health Status Predicting Elder Mistreatment Incidence

	Any Mistreatment Incidence	Neglect Incidence[a]	Emotional Abuse Incidence[a]	Physical Abuse Incidence[a]	Financial Abuse Incidence[a]
Predictors	*OR (95% CI)*	*OR (95% CI)*	*OR (95% CI)*	*OR (95% CI)*	*OR (95% CI)*
Age	1.02 (0.98 – 1.06)	1.17 ** (1.04 – 1.36)	0.97 (0.89 – 1.04)	1.02 (0.94 – 1.10)	1.01 (0.97 – 1.06)
Sex (ref. Male)					
Female	0.62 (0.34 – 1.13)	1.19 (0.21 – 11.63)	0.50 (0.19 – 1.29)	0.36 (0.11 – 1.11)	0.73 (0.35 – 1.56)
Self-rated Health (ref. Good)					
Poor	1.73 ** (1.25 – 2.41)	1.09 (0.34 – 3.35)	2.03 * (1.16 – 3.55)	1.64 (0.90 – 2.99)	1.74 ** (1.17 – 2.60)
Household Income	0.98 (0.84 – 1.15)	0.91 (0.53 – 1.47)	0.94 (0.72 – 1.23)	1.16 (0.86 – 1.60)	0.90 (0.75 – 1.09)
Race/Ethnicity (ref. non-Hispanic/White)					
Black	2.33 * (1.03 – 5.12)	4.55 (0.44 – 42.46)	1.14 (0.26 – 4.01)	1.02 (0.10 – 5.41)	2.46 (0.96 – 6.11)
Hispanic	0.75 (0.11 – 2.93)	2.79 (0.02 – 44.85)	0.42 (0.00 – 3.92)	0.54 (0.00 – 4.56)	1.37 (0.20 – 5.77)
Other Race	1.11 (0.16 – 4.40)	4.56 (0.03 – 61.14)	0.42 (0.00 – 4.33)	0.86 (0.01 – 7.33)	1.98 (0.28 – 8.35)
Geo-Cultural Context (ref. Urban)					
Suburban	0.79 (0.38 – 1.57)	1.15 (0.10 – 9.65)	0.98 (0.30 – 2.87)	0.66 (0.16 – 2.19)	0.68 (0.25 – 1.65)
Rural	0.61 (0.24 – 1.40)	1.72 (0.14 – 17.48)	0.60 (0.14 – 2.07)	0.42 (0.04 – 2.07)	0.79 (0.27 – 2.09)
Co-Residence (ref. Lives Alone)					
Live With Family	0.72 (0.39 – 1.33)	0.91 (0.14 – 6.18)	1.19 (0.42 – 3.82)	0.44 (0.14 – 1.43)	0.62 (0.30 – 1.30)
Functional Impairment	0.88 (0.52 – 1.24)	0.98 (0.52 – 1.58)	0.63 (0.01 – 1.55)	1.07 (0.03 – 1.79)	0.98 (0.60 – 1.38)
Health Status (ref. Stays Same)					
Health Better	1.92 (0.99 – 3.80)	1.02 (0.14 – 7.65)	2.10 (0.69 – 6.57)	1.03 (0.27 – 3.62)	1.91 (0.83 – 4.49)
Health Worse	0.83 (0.38 – 1.78)	0.77 (0.06 – 6.95)	0.86 (0.23 – 2.85)	0.52 (0.10 – 2.02)	1.21 (0.48 – 2.97)
Observations	523	515	509	521	516

OR – Odds Ratios, CI- Confidence Interval, a – Firth's bias reduced logistic regression, $p<0.05$ ** $p<0.01$*

© 2021 Burnes D et al. *JAMA Network Open.*

eTable 4. Multivariable Logistic Regression for Change in Income Predicting Elder Mistreatment Incidence

	Any Mistreatment Incidence	Neglect Incidence[a]	Emotional Abuse Incidence[a]	Physical Abuse Incidence[a]	Financial Abuse Incidence
Predictors	*OR (95% CI)*	*OR (95% CI)*	*OR (95% CI)*	*OR (95% CI)*	*OR (95% CI)*
Age	1.02 (0.98 – 1.07)	1.12 (1.00 – 1.29)	0.97 (0.90 – 1.05)	1.03 (0.95 – 1.12)	1.02 (0.97 – 1.08)
Sex (ref. Male)					
Female	0.59 (0.31 – 1.13)	0.68 (0.09 – 6.93)	0.38 (0.14 – 1.02)	0.39 (0.11 – 1.23)	0.77 (0.35 – 1.74)
Self-rated Health (ref. Good)					
Poor	2.75 * (1.22 – 5.94)	1.69 (0.11 – 11.95)	3.51 * (1.06 – 11.21)	4.65 * (1.21 – 15.93)	2.35 (0.89 – 5.71)
Household Income	0.97 (0.81 – 1.15)	0.86 (0.48 – 1.49)	0.85 (0.65 – 1.12)	1.18 (0.86 – 1.67)	0.86 (0.70 – 1.07)
Race/Ethnicity (ref. non-Hispanic/White)					
Black	2.34 (0.97 – 5.39)	9.06 (0.90 – 127.45)	1.51 (0.35 – 5.17)	1.13 (0.12 – 5.70)	2.17 (0.76 – 5.78)
Hispanic	0.95 (0.14 – 3.77)	4.15 (0.02 – 91.84)	0.48 (0.00 – 4.34)	0.57 (0.00 – 5.03)	1.76 (0.25 – 7.42)
Other Race	1.98 (0.28 – 8.54)	8.80 (0.06 – 167.16)	0.50 (0.00 – 5.82)	1.82 (0.01 – 17.19)	3.26 (0.45 – 15.09)
Geo-Cultural Context (ref. Urban)					
Suburban	0.94 (0.44 – 1.92)	1.75 (0.13 – 23.00)	1.23 (0.38 – 3.64)	0.76 (0.19 – 2.52)	0.71 (0.25 – 1.81)
Rural	0.75 (0.29 – 1.75)	3.05 (0.21 – 50.84)	0.72 (0.17 – 2.50)	0.46 (0.05 – 2.29)	0.88 (0.29 – 2.38)
Co-Residence (ref. Lives Alone)					
Lives With Family	0.70 (0.37 – 1.35)	0.96 (0.11 – 11.68)	1.47 (0.50 – 5.15)	0.43 (0.13 – 1.37)	0.55 (0.26 – 1.19)
Functional Impairment	0.85 (0.47 – 1.24)	1.06 (0.59 – 1.82)	0.88 (0.01 – 1.58)	0.98 (0.02 – 1.66)	0.95 (0.55 – 1.36)
Household Income (ref. Stays Same)					
Becomes Lower	1.92 (0.94 – 4.06)	3.56 (0.56 – 46.45)	2.07 (0.66 – 7.63)	1.29 (0.33 – 5.54)	2.14 (0.88 – 5.56)
Becomes Higher	1.45 (0.65 – 3.23)	0.60 (0.00 – 13.44)	1.27 (0.35 – 4.88)	1.93 (0.48 – 8.83)	1.56 (0.60 – 4.16)
Observations	462	457	450	461	458

OR – Odds Ratios, CI- Confidence Interval, a – Firth's bias reduced logistic regression, p<0.05 ** p<0.01 *** p<0.001*

© 2021 Burnes D et al. *JAMA Network Open.*

eTable 5. Multivariable Logistic Regression for Change in Functional Capacity Predicting Elder Mistreatment Incidence

	Any Mistreatment Incidence[a]	Neglect Incidence[a]	Emotional Abuse Incidence[a]	Physical Abuse Incidence[a]	Financial Abuse Incidence[a]
Predictors	*OR (95% CI)*	*OR (95% CI)*	*OR (95% CI)*	*OR (95% CI)*	*OR (95% CI)*
Age	1.01 (0.97 – 1.06)	1.13 (1.00 – 1.34)	0.97 (0.90 – 1.05)	1.02 (0.94 – 1.11)	1.01 (0.96 – 1.06)
Sex (ref. Male)					
Female	0.62 (0.34 – 1.11)	1.17 (0.20 – 11.19)	0.47 (0.18 – 1.22)	0.38 (0.11 – 1.18)	0.71 (0.35 – 1.48)
Self-rated Health (ref. Good)					
Poor	2.59 * (1.22 – 5.25)	1.63 (0.08 – 13.62)	4.04 * (1.26 – 11.75)	4.16 * (1.14 – 13.47)	2.49 * (1.02 – 5.67)
Household Income	0.96 (0.82 – 1.12)	0.93 (0.57 – 1.41)	0.90 (0.70 – 1.17)	1.17 (0.87 – 1.62)	0.88 (0.73 – 1.05)
Race/Ethnicity (ref. non-Hispanic/White)					
Black	2.41 * (1.08 – 5.19)	4.13 (0.41 – 38.83)	1.47 (0.34 – 5.19)	1.15 (0.12 – 6.19)	2.59 * (1.03 – 6.26)
Hispanic	0.92 (0.17 – 3.19)	1.69 (0.01 – 32.52)	0.39 (0.00 – 3.39)	0.60 (0.00 – 4.99)	1.66 (0.30 – 6.07)
Other Race	1.43 (0.27 – 5.08)	4.08 (0.03 – 59.35)	0.46 (0.00 – 5.29)	1.08 (0.01 – 9.42)	2.52 (0.46 – 9.34)
Geo-Cultural Context (ref. Urban)					
Suburban	0.80 (0.40 – 1.55)	1.10 (0.10 – 8.76)	1.04 (0.33 – 3.02)	0.64 (0.16 – 2.12)	0.73 (0.28 – 1.71)
Rural	0.67 (0.28 – 1.49)	2.21 (0.17 – 23.12)	0.67 (0.16 – 2.27)	0.42 (0.04 – 2.08)	0.85 (0.30 – 2.16)
Co-Residence (ref. Lives Alone)					
Lives With Family	0.74 (0.41 – 1.35)	0.95 (0.14 – 6.94)	1.40 (0.49 – 4.59)	0.46 (0.14 – 1.52)	0.63 (0.31 – 1.28)
Functional Impairment	0.97 (0.62 – 1.43)	0.91 (0.51 – 1.71)	1.08 (0.04 – 1.82)	1.05 (0.03 – 1.80)	1.10 (0.71 – 1.69)
Functional Capacity (ref. Stays Same)					
Becomes Better	1.33 (0.69 – 2.48)	3.67 (0.53 – 42.67)	0.56 (0.13 – 1.89)	0.83 (0.19 – 2.90)	1.24 (0.56 – 2.64)
Becomes Worse	0.57 (0.00 – 6.63)	15.41 (0.02 – 583.28)	1.33 (0.01 – 172.70)	1.71 (0.01 – 158.30)	0.44 (0.00 – 8.04)
Observations	522	515	509	521	516

*OR – Odds Ratios, CI- Confidence Interval, a – Firth's bias reduced logistic regression, * p<0.05*

© 2021 Burnes D et al. *JAMA Network Open.*

eTable 6. Multivariable Logistic Regression for Change in Living Arrangements

	Any Mistreatment Incidence	Neglect Incidence[a]	Emotional Abuse Incidence[a]	Physical Abuse Incidence[a]	Financial Abuse Incidence
Predictors	*OR (95% CI)*	*OR (95% CI)*	*OR (95% CI)*	*OR (95% CI)*	*OR (95% CI)*
Age	1.01 (0.97 – 1.06)	1.17 * (1.03 – 1.51)	0.96 (0.88 – 1.03)	1.03 (0.95 – 1.12)	1.01 (0.96 – 1.06)
Sex (ref. Male)					
Female	0.58 (0.32 – 1.07)	1.64 (0.23 – 33.16)	0.45 (0.17 – 1.18)	0.42 (0.12 – 1.32)	0.66 (0.31 – 1.41)
Self-rated Health (ref. Good)					
Poor	2.70 ** (1.26 – 5.57)	0.66 (0.02 – 23.71)	3.53 * (1.08 – 10.72)	4.30 * (1.16 – 13.88)	2.55 * (1.03 – 5.89)
Household Income	0.98 (0.83 – 1.15)	0.98 (0.51 – 1.65)	0.91 (0.70 – 1.19)	1.14 (0.84 – 1.57)	0.89 (0.74 – 1.08)
Race/Ethnicity (ref. non-Hispanic/White)					
Black	2.66 * (1.16 – 5.95)	9.65 (0.43 – 255.09)	1.30 (0.29 – 4.53)	1.21 (0.12 – 6.55)	2.94 * (1.12 – 7.45)
Hispanic	0.83 (0.12 – 3.24)	5.59 (0.01 – 279.37)	0.45 (0.00 – 3.98)	0.52 (0.00 – 4.51)	1.50 (0.22 – 6.31)
Other Race	1.44 (0.22 – 5.69)	3.78 (0.02 – 89.97)	0.51 (0.00 – 5.26)	0.97 (0.01 – 8.21)	2.57 (0.37 – 10.70)
Geo-Cultural Context (ref. Urban)					
Suburban	0.77 (0.37 – 1.53)	0.89 (0.06 – 9.56)	0.99 (0.31 – 2.87)	0.66 (0.16 – 2.20)	0.68 (0.25 – 1.65)
Rural	0.66 (0.26 – 1.52)	1.68 (0.11 – 21.96)	0.69 (0.17 – 2.34)	0.41 (0.04 – 2.07)	0.86 (0.29 – 2.25)
Live With Family	0.60 (0.29 – 1.24)	0.11 (0.00 – 3.73)	1.11 (0.33 – 4.24)	0.63 (0.17 – 2.33)	0.45 (0.19 – 1.05)
Functional Capacity	0.90 (0.53 – 1.28)	1.21 (0.62 – 2.70)	0.81 (0.02 – 1.62)	0.94 (0.02 – 1.64)	1.01 (0.62 – 1.42)
Living Arrangements (ref. Stays Same)					
Alone to With Others	1.31 (0.38 – 3.94)	3.21 (0.21 – 42.97)	1.33 (0.12 – 8.15)	1.36 (0.13 – 7.84)	0.92 (0.19 – 3.40)
With Others to Alone	2.27 * (1.00 – 5.00)	36.47 * (1.88 – 3393933.37)[b]	1.84 (0.48 – 6.16)	0.25 (0.00 – 2.31)	2.74 * (1.01 – 7.21)
Observations	523	515	509	521	516

*OR – Odds Ratios, CI- Confidence Interval, a – Firth's bias reduced logistic regression, b – Due to large CI, result should be considered non-significant and disregarded, * p<0.05 ** p<0.01*

© 2021 Burnes D et al. *JAMA Network Open.*
